# Age related variations observed in the oral microbiome of wild wood mice, *Apodemus sylvaticus*

**DOI:** 10.1080/20002297.2017.1325271

**Published:** 2017-05-30

**Authors:** Susan Joseph, Asil Aslam, Andrew Cunningham, Mike Curtis

**Affiliations:** ^a^ Barts and The London Institute of Dentistry, Queen Mary University of London, London E1 2AD, UK; ^b^ FERA Science, Sand Hutton, York, UK

## Abstract

We compared the oral microbiome of wild wood mice (*Apodemus sylvaticus*) at four ages (18, 22, 26 and 30 weeks). 25 wood mice captured and bred at FERA Science were swabbed orally and the microbial population was characterized by aerobic and anaerobic culture methods followed by 16S rRNA sequencing. The total oral bacterial counts in the wild mice were found to be ~10^7^ cfu/m^l^ among all age groups, which was 10-fold higher than the counts in our laboratory “specific pathogen free” (SPF) mice. The predominant genera identified in the wood mice were *Pasteurella, Lactobacillus, Neisseria* and *Streptococcus*, present at varying proportions across ages. Organisms belonging to *Pasteurella* and *Neisseria* were found to increase with the progression of age, while a 20% drop was observed among the members of the *Streptococcus* genus in 30w old mice compared to the younger 18w old animals. Alveolar bone loss analyses of the jaws of these mice is also being currently carried out, along with the analysis of the oral and faecal microbiome using next generation sequencing methods. Comparison of these results with age matched laboratory mice can aid in understanding the influence of the natural environment on oral health development in mice.

